# News: Agencies

**Published:** 2011-06

## The Bill and Melinda Gates Foundation

### Bill Gates calls for prioritizing vaccines at the 64th World Health Assembly

Bill Gates addressed government leaders at the recent World Health Assembly, which is the highest-level decision-making body of the World Health Organization. He called upon them to increase funding support for global vaccine roll-out, also stressing their accountability in providing the large benefits of vaccination to every child. He also called for this support to be shown at the GAVI Alliance pledging meeting in London in June 2011. Vaccines remain the most cost-effective interventions in averting the deadly burden of childhood infections; in recent years, Bill Gates has assumed a role of the most prominent advocate who ensured that vaccines were given proper attention and funding support. The most recent estimates show that the GAVI Alliance’s vaccination roll-out has resulted in several millions of child deaths prevented over the past decade.

### Bill Gates calls for polio eradication, United Kingdom responds positively

In early 2011, Bill Gates called the global health community for a final push, which should see polio eradicated. This crippling disease is hardly remembered in wealthy countries, but there are still areas of the world where the vaccine has not reached children and where pockets of disease persist. Bill Gates launched his call in a Manhattan town house that was once a property of possibly the most highly profiled victim of this disease – Franklin D. Roosevelt. The push to eradicate polio can be dated back to 1985, and the Bill and Melinda Gates Foundation started their large investments towards this goal in 2005. The Foundation’s donations have changed the context and they have now emerged as the key donor – having invested up to US$ 1.3 billion (€ 0.9 billion). British Prime Minister David Cameron has responded positively, announcing that the United Kingdom would double its contributions to polio eradication.

### Smallpox eradication pioneer expresses scepticism over Gates’ polio goal

Donald Henderson is an acclaimed pioneer of smallpox eradication. Having heard Bill Gates’ call for polio eradication, he expressed some scepticism over the feasibility of this goal. As reported by the Financial Times, Donald Henderson said that polio eradication had “...become more of a ‘movement’ than a public health initiative capable of being examined by objective judgment.” In his opinion, polio may not be “susceptible” to eradication in the same way as other infectious diseases have been in the past. He sees the future of the fight against polio in cheaper, but sustained control programmes with regular annual immunisations, which should minimize the devastating effects of polio over time.

### Gates Foundation announces winners of Grand Challenges Exploration grants, launches Round 7

The Bill & Melinda Gates Foundation have been praised for their remarkable grant-awarding scheme. They provide US$ 10 000 (€ 69 407) to each of the large number of recipients who propose to start-up innovative research with potential to improve lives in poor countries. This unique funding scheme, called “Grand Challenges Exploration” (GCE), offers a total of US$ 100 million (€ 69 million) to the awardees. It has been designed to encourage innovation and enable creative researchers worldwide to test genuinely novel ideas that could address persistent global health issues in an effective way. A total of 88 new winners were announced after Round 6 of these grants had been closed. The Foundation has been accepting proposals for Round 7 of the scheme up until late May 2011. The new calls focus yet again on research areas where unconventional thinking is needed. The topics in Round 7 included creating ways to accelerate, sustain and monitor polio eradication; creating the next generation of sanitation technologies; creating low-cost cell phone-based solutions for improved uptake and coverage of childhood vaccinations; designing new approaches to cure HIV infection; exploring nutrition for healthy growth of infants and children; and applying synthetic biology to global health challenges.

### WHO’s programme for research and training in tropical diseases wins the 2011 Gates Award

This year’s Gates Award for Global Health has been given to the Special Programme for Research and Training in Tropical Diseases (TDR). This programme, hosted and co□nanced by the headquarters of the World Health Organization in Geneva, has also been funded by UNICEF, UNDP and the World Bank. One of the world’s largest public health prizes was awarded to programme Director Robert Ridley at a ceremony in Washington, USA. Over the past 36 years, the programme has attracted researchers from all over the world who worked with TDR to find improved health solutions for people in poor countries, resulting in major progress against many infectious diseases of the poor.

## The GAVI Alliance (formerly The Global Alliance for Vaccines and Immunisation)

### GAVI raises US$ 4.3 billion, exceeds targets and meets its □nancial needs until 2015

The global vaccine charity – Global Alliance for Vaccines and Immunisation (GAVI) – held a donor summit in London on June 13th in a bid to overcome its financial shortfall and secure its financial needs for the next several years. International donors eventually pledged US$ 4.3 billion (€ 3 billion), which will be used to vaccinate nearly 250 million children against the leading causes of child deaths such as pneumonia and diarrhoea. The donors which were critical for this success, which far exceeded gloomy expectations, were the governments of the United Kingdom, Norway and the Bill & Melinda Gates Foundation. The United Kingdom pledged US$ 1.34 billion (€ 0.9 billion), the Gates Founation promised US$ 1 billion (€ 0.7 billion) and Norway offered US$ 677 million (€ 463 million). This should allow GAVI to carry out all its immunization plans through 2015.

### Ten years of GAVI Alliance – Progress Report 2010

Earlier this year GAVI has marked 10 years of its operations. It has also appointed a new Chief Executive Officer (CEO), Seth Berkley, who has been a founder and CEO of the International AIDS Vaccine Initiative. His strong track record as a global vaccines advocate has been praised and his appointment widely welcomed. GAVI has also issued its progress report for the year 2010. Some of the highlights include an estimate of more than 5 million future child deaths that have been prevented through GAVI activities. GAVI also estimated that more than 288 million additional children had been immunised with support from GAVI and its partners. Recently, GAVI are increasingly focusing on vaccines that could prevent childhood pneumonia and diarrhoea – the leading killers. It is hoped that this could contribute to preventing further 4 million deaths by 2015.

### Large pharmaceutical companies finally reduce the prices of vaccines for the poor

In June 2011, leading pharmaceutical companies that produce vaccines against childhood infections announced that they will reduce the costs of their products for the poorest countries by a truly substantial amount. GlaxoSmithKline (GSK), Merck, Johnson & Johnson and Sanofi-Aventis have all agreed to cut prices through the international vaccine alliance, GAVI. This welcome move comes after years of pressure and calls on these companies to consider this reduction in order to enable life-saving vaccines to reach the low resource settings, where child deaths from the preventable infectious diseases tend to cluster. The vaccines included in this strategic decision by the companies will protect against diarrhoea (ie, rotavirus) and human papillomavirus. A pentavalent vaccine which prevents diphtheria, tetanus, pertussis, hepatitis B, and Haemophilus infiuenzae type b will also be offered at a dramatically reduced price. GlaxoSmith-Kline will supply developing countries with its vaccine against rotavirus at a 95 per cent discount to the western market price.

### Non-governmental organizations unhappy with appointment of a drug company to GAVI board

Although the GAVI Alliance continues to make remarkable progress towards immunizing children world-wide and preventing diseases and deaths, it is not free of critics. There are several non-governmental organizations (NGOs) which continue to stress that GAVI serves well the interests of large pharmaceutical companies, too, and that it has not done enough to improve the value for the money which GAVI donors are currently paying for the life-saving vaccines. They demand to see the prices of those vaccines lowered substantially. Earlier this year a Dutch pharmaceutical company Crucell (recently acquired by Johnson & Johnson) has been appointed to the board of the GAVI Alliance, which again sparked NGO’s “... concerns over conflicts of interest and demands for tougher competition to reduce prices,” according to the Financial Times. This is because nearly 60% of Crucell’s revenues in 2010 were coming from sales of its pentavalent vaccine to GAVI.

### GAVI strengthened by the former CEO of MTV networks

The Global Alliance for Vaccines and Immunisation (GAVI) has announced that Bill Roedy, who was the key developer of the MTV Networks, has agreed to join the GAVI network and assist their advocacy for immunisation world-wide. Bill Roedy, former CEO of the MTV, will become the first GAVI Envoy. This unexpected appointment underscores the importance of the public perception of the value of immunization. The recent scare over (apparently unfounded) reports that some vaccines may be linked to autism in children has revealed how quickly the behaviour of the general public changes under the influence of media reports, regardless of their accuracy. Mr Roedy is expected to raise awareness about the importance of vaccines, especially in fighting the main killers of children globally – such as pneumonia and diarrhoea.

## The World Bank

### Shifting the funding for global health from vertical to horizontal

As recently discussed in the *New England Journal of Medicine*, health systems researchers have long debated whether health care is better organized ‘vertically’ or ‘horizontally’. Vertical funding usually refers to supporting one (or a few) specific diseases, while horizontal funding can affect many diseases at the same time, through supporting health care systems. Examples of vertical interventions in global health are eg, smallpox and polio immunization programmes. Horizontal approaches include eg, support to primary care, as advocated by the World Health Organization’s 1978 Alma Ata Declaration, or sector-wide approaches to promoting health care reform, supported by the World Bank. The US President’s Emergency Plan for AIDS Relief (PEPFAR) and the Global Fund to Fight AIDS, Tuberculosis, and Malaria are further examples of disease-specific funding initiatives. Although many theoretical models predict larger benefits from horizontally structured support, there are very few such programs in place. In the world of global health today, it is much easier to get donors enthusiastic about the more specific, vertical programs.

### Innovative health financing – the role of Advance Market Commitment

According to Wikipedia, an ‘advance market commitment’ (AMC) is “...a binding contract, typically offered by a government or other financial entity, used to guarantee a viable market if a vaccine or other medicine is successfully developed. As a result of such a commitment, the market for vaccines or drugs for neglected diseases would be comparable in size and certainty to the market for medicines for rich countries. This would enable biotech and pharmaceutical companies to invest in the development of new vaccines to tackle the world’s most pressing health problems, such as pneumonia, diarrheal disease, HIV/AIDS and malaria, in the normal course of their business decisions.” An editorial in *Lancet Infectious Diseases* recently described how the roll-out of the GAVI initiative is helping to provide poor countries with low-cost pneumococcal vaccines: “The recent launch of pneumococcal vaccination in Nicaragua under AMC has shown that innovative approaches to health financing can benefit both global health and pharmaceutical companies.”

### Center for Global Development publishes an analysis of the future of development finance

In a recently published ‘Working Paper 250’, posted online by the Center for Global Development (which conducts independent research and develops practical ideas for global prosperity), it has been stressed that development finance is currently “at a turning point.” The report mentions a “triple revolution of goals, actors and tools.” The report predicts that “...as much of Asia grows its way out of poverty, aid will increasingly be focused on Africa and on countries plagued by instability, or with governments unable to meet the basic needs of their populations.” Also, the share of development finance directed to tackling global public goods – like climate change, conflict prevention, and public health – is likely to expand substantially. The authors predict that the responsibility for addressing global challenges will increasingly be borne by coalitions that cut across States, the private sector and civil society. It sees the role of multilaterals (such as WHO, GAVI, Global Fund, UNICEF, UNAIDS and the World Bank) as focused on providing a coordinated mechanism/platform for delivering common objectives.

### World Bank identifies five poor African states as potential “Growth Poles”

Africa is lagging behind the rest of the world in most economic and health indicators. However, this presents the continent with a remarkable opportunity for growth and development over the coming decades, at the rate which could hardly be expected anywhere else, according to the World Bank. Its new strategy for the continent aims to leverage growing South-South investments, which have recently been initiated by the growing low and middle income economies like China, India, Brazil and South Africa. The World Bank would like to ensure more inclusive development. To foster this development strategy, the World Bank suggested five poor states as ‘Growth Poles’ of the new Africa. These poles are being planned in Madagascar, Cameroon, Mozambique, The Gambia and the Democratic Republic of Congo.

### Pakistan seeks financial aid from the World Bank to purchase polio vaccine

The government of Pakistan has been reported to request an emergency financial package worth US$ 41 million (€ 28 million) from the World Bank to purchase oral polio vaccine. The move should support its polio eradication initiative. The financial package should be awarded by the International Development Association of the World Bank. This assistance to the government of Pakistan is given under the third project since the year 2003, enabling it to procure OPV as part of the larger global campaign. The aid should help Pakistan to meet this year’s vaccine requirements in support of the National Emergency Action Plan 2011 for polio eradication in the country.

## United Nations (UN)

### High-level UN meetings to focus on burden of non-communicable diseases

In January 2011, Ban Ki-moon called on the world’s business leaders to help address the risk factors which underlie the most prevalent non-communicable diseases (NCDs). Health ministers from many countries met under the auspices of the United Nations in late April 2011 to adopt tougher measures against non-communicable diseases. These measures include preventive approaches, such as the promotion of healthy lifestyles and encouraging a multi-sectoral approach to prevention and treatment of NCDs. NCDs, principally heart disease, stroke, cancer, diabetes, and chronic respiratory diseases, have recently emerged as the leading cause of morbidity and mortality not only in high-income countries, but also in low and middle income countries. The UN High-Level Meeting (UN HLM) on NCDs in September 2011 will present global leaders with an opportunity to develop a coordinated global response to NCDs.

### General Assembly appoints Ban Ki-moon to second term as UN Secretary-General

On 21st June 2011 the United Nations General Assembly agreed to appoint Ban Ki-moon to a second consecutive term as the Secretary-General of the Organization. Under the resolution, which was adopted by acclamation, his second term will run until the end of 2016. He is the eighth person to serve as UN chief and has been in office since January 2007. Following the re-appointment, Ban Ki-moon told the Assembly that he was “proud and humbled to accept it.” He also said that the UN had “laid a firm foundation for the future” on a number of issues since he assumed office, including climate change, nuclear disarmament, education, sustainable development and global health.

### WHO highlights the growing burden of non-communicable diseases

In April 2011, the World Health Organization reported that chronic illnesses of late onset (such as cancer, heart disease and diabetes) now cause more deaths than all other diseases combined. WHO, the United Nations’ health body, issued a global report on non-communicable diseases (NCDs). NCDs have reached epidemic proportions and pose a much greater threat world-wide than infectious diseases. They caused about 63% of the 57 million deaths recorded globally in 2008. Nearly 80% of these deaths were in low and middle income countries. Furthermore, NCDs are also projected to rise further in the coming decades, especially in rapidly growing middle income countries. Cost-effective interventions, such as reducing risk factors, early detection and timely treatment, will become critical in tackling the problem. However, the capacity of many low and middle income countries to implement those interventions is poor.

### United Nations are increasingly harnessing the power of social media

The United Nations Department of Public Information (DPI) is increasingly using social media and the internet to disseminate the work of the United Nations, according to UN News. In April 2011, Kiyo Akasaka, Under-Secretary-General for Communications and Public Information, told the opening of the latest session of the UN’s Committee on Information that the recent popular uprisings across North Africa and the Middle East illustrated the power and reach of social media tools. Furthermore, in an effort to encourage individuals to help feed tens of thousands of hungry children across the world, the United Nations World Food Programme (WFP) has created a social media platform through which people can make donations to the agency.

### Tracking the progress towards UN’s Millennium Development Goals

Each year the World Health Organization presents a report summarizing the state of health in its 193 member countries. Part of the report usually focuses on tracking the progress towards the UN’s Millennium Development Goals (MDGs). These goals, set in 2000 by consensus of all member states, aimed to ensure political commitment to accelerating global progress in health and development. This year’s report shows continuing overall progress, but there are still regions where little or no improvement has been made. Child mortality in some countries in sub-Saharan Africa is now higher than it was in 1990, while the fourth MDG demands a reduction by two-thirds between 1990 and 2015. In parallel, the World Bank estimated that two-thirds of developing countries are either on track, or very close, to meeting key targets for tackling extreme poverty and hunger.

### UN Secretary General calls world leaders to end AIDS by 2020

Ban Ki-moon used the three-day UN’s summit on AIDS in June 2011 to call upon world leaders to end AIDS by 2020. He said: “That is our goal – zero new infections, zero stigma and zero AIDS-related deaths.” The meeting in New York marked the 30th anniversary of the discovery of human immunodeficiency virus (HIV). According to UN’s estimates, some 34 million people have AIDS, but up to 50% do not know that they have the disease. More than 9 million people still do not get antiretroviral treatment, and about 1.8 million people die from AIDS each year. Ban Ki-moon stressed that new infections have dropped by 20% since 2001, when the world leaders first organized and developed plans to control the pandemic. The meeting was attended by 30 presidents and heads of government, and African leaders spoke of the desperate need for more financial support to fight the disease in their nations.

### UN AIDS summit aims to cover 15 million sufferers with medical treatment

A UN AIDS summit this year has set a target of more than doubling the global coverage of life-saving AIDS treatment. The concrete target is to provide 15 million AIDS patients with antiretroviral treatment by 2015. Over the past three decades, the number of cases increased from a small group of young homosexual men in Los Angeles to 34 million men, women and children globally, who are estimated to be living with HIV today. It is hoped that, within the next decade or two, this trend can be reversed and AIDS stopped through a combination of high coverage of antiretroviral drugs and the development of a vaccine that will prevent the disease.

### Cheaper antiretroviral drugs will be offered to 70 of the world’s poorest countries

Although cocktails of AIDS drugs which once cost more than US$ 10 000 (€ 6905) per year in wealthy countries are now available in poor countries for less than US$ 200 (€ 138), many patients still receive drugs which were developed decades ago and can have very serious side effects. The World Health Organization now recommends combinations containing tenofovir because they have fewer side effects and show less risk of development of resistance by the virus. The current price of one such combination is US$ 159 (€ 110), which is down from about US$ 400 (€ 278) three years ago. The lower prices of these drugs will be offered by eight Indian pharmaceutical companies. They were negotiated by the Clinton Health Access Initiative, with support from British foreign aid and the Bill and Melinda Gates Foundation. The funding which will guarantee the initial payments, and which is crucial to getting the Indian suppliers to increase the production of these drugs, will come from Unitaid. Unitaid is an independent agency founded at the United Nations, which collects several agreed taxes which were set up to finance global health programs.

### Babies who escape mother-to-child HIV transmission are at increased risk of other diseases

According to a study published by *JAMA*, babies who manage to escape mother-to-child HIV transmission still face up to a four times greater risk of dying in the first year of life. The main reason is thought to be a greater susceptibility to infectious disease. Researchers examined some 100 mothers and babies in South Africa and compared antibody levels among children who were born infected with HIV to children who escaped HIV. Those who did not get HIV showed lower levels of antibodies to whooping cough, tetanus and Pneumococcus infections. All those infections are vaccine-preventable, but vaccines are not always available to these children. Births of HIV-positive babies have dropped dramatically in the past decade due to use of medications that mothers can take during pregnancy to prevent transmission.

## UNICEF

### China reports large successes in AIDS mortality reduction

Government scientists in China have reported that AIDS mortality in the country has decreased by nearly two-thirds since free antiretroviral drugs were introduced in 2002. It is estimated that the coverage by AIDS drugs among those who need it in China has reached about 63%, while it was practically non-existent in 2002. Consequently, a 64% drop in mortality (measured per 100 ‘person-years’) is reported: from 39.3 in 2002 to 14.2 in 2009. The study, which was led by China’s national Center for control and prevention of AIDS and other sexually transmitted diseases, was published by Lancet Infectious Diseases. The number of infected people in China has reached nearly 750 000, but in a population of 1.3 billion the prevalence is still less than 1 in 1000 population. Of those infected, it is estimated that more than 300 000 have been tested and more than 80 000 are being treated. China begins treatment when a patient’s CD4 cell count (which is a measure of immune system strength) drops below 350 per cubic millimetre. The government’s experts are now debating whether to start treatment as soon as a patient tests positive for HIV. This strategy, also known as ‘treatment as prevention’, can reduce the risk of new infections by up to 96%, because it protects sexual partners.

### UNICEF’s ‘State of world’s children’ report for 2011 focused on adolescents

The key theme of UNICEF’s ‘State of world’s children’ (SOWC) report for the year 2011 is investing in adolescence to break cycles of poverty and inequity. Today, young people throughout the world face the problems of economic turmoil, climate change, environmental degradation, urbanisation, migration and the rising costs of health care. Strong investments during the last two decades have resulted in large progress in the health and welfare of young children, but there have been fewer gains in areas critically affecting adolescents. Anthony Lake, UNICEF Executive Director, said that “...adolescence is a pivot point – an opportunity to consolidate the gains we have made in early childhood or risk seeing those gains wiped out.” In the report, UNICEF says investment is needed to improve data collection to increase the understanding of adolescents’ problems; invest in education and training to help adolescents lift themselves out of poverty; expand opportunities for youth to participate and voice their opinion; promote laws, policies and programmes that protect the rights of adolescents; and prevent poverty and inequity.

### UNICEF’s initiative to promote transparency in vaccine pricing

Many stakeholders in the global health community have been expressing concern in recent years that large pharmaceutical companies are using the global health funding drive to generate huge profits on life-saving vaccines. They argued that vaccines against life-threatening childhood infections should be made available for the poor at much reduced prices, and profits reduced. UNICEF launched an initiative recently to improve transparency by making vaccine prices available on its Web site. For the first time, UNICEF publicly listed the prices it pays individual drug manufacturers for vaccines. It is hoped that this move will lead to a more competitive market and lower prices, while the donors will also be assured that UNICEF and GAVI are getting reasonable prices. UNICEF has traditionally been one of the largest buyers of children’s vaccines and this move should ensure that vaccine supply is sustainable and affordable. UNICEF’s partners in immunisation, such as GAVI Alliance, welcomed this positive development.

### UNICEF carries out yellow-fever immunization, supports restoring education in Côte d’Ivoire

Although the country has been facing political uncertainties and concerning unrests in aftermath to the 2010 presidential elections, UNICEF carried out immunization against yellow fever among nearly 1 million people in this troubled country. This move was conducted in four health districts with UNICEF’s support as a result of 25 reported deaths from the disease since November 2010. UNICEF’s Officer-in-Charge for Côte d’Ivoire, Sylvie Dossou, expressed gratitude to the GAVI Alliance for providing the yellow-fever vaccines, and to the World Health Organization for their partnership with UNICEF in carrying out this critically important campaign. In addition, UNICEF assisted more than 1 million children to return to school after months of disruption to the country’s education system, caused by the political and security crisis in the country.

### Online consultation process launched to set standards for child-friendly businesses

Together with Save the Children and UN Global Compact, UNICEF has launched an online consultation process. The aim is to invite businesses and civil society to take an active role in developing a global standard of business principles pertaining to children’s rights. An online consultation process should enable representatives of the private sector and civil society to shape the Children’s Rights and Business Principles, setting the standard for child-friendly businesses everywhere. A series of follow-up meetings and global consultations is planned. The first meeting will take place in London, and it is hoped that it will attract leading business and civil society representatives.

### On the 21st annual Day of the African Child, UNICEF reminds governments to protect children

A very large number of children in Africa still experience violence, exploitation and abuse – many of them on a daily basis. This problem is particularly troublesome among children who live and work on the streets of Africa. Recently, on the occasion of the 21st annual Day of the African Child, UNICEF called on African governments to strengthen support systems which can provide a more protective environment in families and communities to keep children safe. The main approach is to strengthen families through the provision of basic social, health and education services.

## World Health Organization (WHO)

### World Health Organization undergoes a major reform

The last World Health Assembly – the highest-level decision-making body at the World Health Organization (WHO) – has supported proposed reforms which could bring the most substantial changes to the agency in more than six decades of its history. The reforms are clearly needed at WHO. Only a decade ago, it was the only important agency focused on global health issues. Today, it is struggling to maintain its relevance amidst the surge of sharply focused, well managed, innovative, and better funded big new players, such as The Global Fund, the GAVI Alliance and the Bill & Melinda Gates Foundation. Global health issues have attracted an unprecedented interest among the donors over the past decade, but WHO has not seen much of these funds. In fact, WHO reported a US$ 300 million (€ 209 billion) deficit in 2010. The reforms at WHO will see the agency slashing its next budget by nearly US$ 1 billion (€ 0.7 billion) and cut hundreds of jobs at the Geneva headquarters and elsewhere at regional offices. The agency’s Director General, Margaret Chan, explained these cuts as being due to financial problems among rich donor nations and the exchange rate for the weak US dollar. Addressing the annual assembly, she also said WHO was clear of suspicion of pharmaceutical industry influence on the management of the H1N1 pandemic, and that innovative financing mechanisms from the GAVI Alliance had helped to introduce new vaccines against childhood pneumonia and diarrhoea to developing countries.

### WHO: fighting for relevance in the new world of global health

After long consultations with WHO member states on its funding support, Director-General Margaret Chan called the WHO overstretched and unable to respond with sufficient speed and efficiency to many global health problems. More than six decades ago, the United Nations (UN) granted the WHO extensive normative powers which established this agency as the only relevant authority on international health globally. However, several modern initiatives, such as the Global Fund to Fight AIDS, Tuberculosis and Malaria, the GAVI Alliance (formerly the Global Alliance for Vaccines and Immunisation), US President’s Emergency Plan for AIDS Relief (PEPFAR) and well-funded donor agencies (such as the Bill & Melinda Gates Foundation) established themselves as the new leaders in their respective fields of interest. These agencies do not suffer from constraints which often interfere with WHO’s efficiency. Now, WHO is facing a financial crisis, at a time when funding support for global health issues has never been greater. Many donors question the WHO’s actual performance, find the new initiatives a better and safer investment, and worry about the WHO’s vision, efficiency and focus. The existing funding support for WHO largely targets its extra-budgetary activities, while there seems to be little enthusiasm for supporting the WHO’s core budget, which is under the tight political control of its leaders.

### An attempt by the WHO to explore the issue of fake medicines will require more time

Fake drugs, or counterfeit medicines, are beginning to pose an increasingly serious threat to global public health. It is estimated that up to 15% of all medicines that are being sold worldwide are fake. This problem has attracted attention at the World Health Assembly in 2010. The member states requested WHO to establish an intergovernmental working group on counterfeit medicines which will decide WHO’s role in tackling this problem. According to the journal *The Lancet*, although this intergovernmental group was required to make specific recommendations to this year’s 64th World Health Assembly in May 2011, they only met once (in February 2011) and agreed that they needed more time.

### WHO’s careful warning on mobile phone link to brain cancer

In June 2011, the World Health Organisation issued a statement which was quickly disseminated through the global media. The agency warned that there was some evidence linking use of mobile phones to brain cancer. But this warning was worded very carefully, and rightly so. Most human diseases are hugely multi-factorial and caused by an interaction of many risk factors and their interplay with the genetic susceptibility of the host. Because of this, contributions of individual risk factors to an overall burden of disease in the population are typically rather small, albeit real. Because of this general property of most human diseases, it is wise not to overplay the role of individual risk factors, such as mobile phone use, when reporting these disease associations to the media. The story could have potentially been very damaging to the thriving mobile phone industry, but a well-balanced warning from the WHO will be unlikely to cause such damage.

### WHO to address innovative financing of research and development for the poor

The WHO has established a Consultative Expert Working Group on Research and Development: financing and coordination (CEWG). The group met in April 2011 to define their mandate and work plan. One of the main tasks of this group will be to assess proposals for innovative financing of research and development which should serve the needs of the people in low resource settings. CEWG launched a call for proposals and ideas for innovative financing, which will be posted on the CEWG Web site. CEWG plans to analyse the proposals and submit its report to the World Health Assembly in 2012.

